# Reviews, Notices, &c.

**Published:** 1857-11

**Authors:** 


### REVIEWS, NOTICES, &c.

*Littell's Living Age*, No. 701, contains an able notice of Bishop Berkeley's Works. *Graham* offers an inviting programme for 1858. *Putnam* has been merged in *Emerson*, opening with "Up the Mississippi," characteristically illustrated: then follows the "Life of Washington," &c., &c., with his Autographs. "Annual Register of Rural Affairs, an Almanac for 1858," by Luther Tucker & Son, Albany, N. Y., 25 cents, with 130 engravings, is well worth a place in every thrifty farmer's home, and still more needed in the unthrifty—for it is full of valuable information. "*Blackwood*, in its forty-fifth year;" "Military Education;" "The Book and the Rocks;" "Scenes of Clerical Life;" "New Sea-Side Studies," &c.

Nostrums.—*Life Illustrated*, published weekly in New York, by Fowlers & Wells, after saying of the *Independent* that it "contains matters of science, literature, art, and information, which constitute it one of the most valuable newspapers published, its reports as to financial and business matters are taken as authority in Wall street," calls in question its morality, by adding: "*It will* publish advertisements of quack medicines and disguised liquor shops. Don't do that friends, and we will put you down as just about right."

When a religious newspaper publishes a communication which it knows to be a lie, and for pay, it places itself in a false position. It sells its principles for a mess of pottage.

A Lady Connoisseur says, that Gail Borden's condensed milk makes a better cup of Tea than any cream that can be procured in New York, and that if used even with brown sugar, the tea is better than when ordinary milk and loaf sugar are used.

That ugly-looking, coarse-papered, bad-printed monthly, of sixteen pages, the South-Western School Journal, 50 cents a year, edited by the Rev. Mr. Heywood, and Noble Butler, of Louisville, Ky., is not surpassed in the universal excellence of its contents by any educational periodical in this country. If it does not pay well enough to be got up, even in the plain style of our own Journal, it is a disgrace to every Kentuckian and to every family of the great "South-west."

The Anatomical Museum, under the control of Dr. Rentz, Broadway, consisting of 400 models, may be profitably visited by every parent in New York. Open to ladies only on Fridays.

Fuel.—Our city readers who have not laid in their coal and wood for winter are assured, that by dealing with Truslow Brothers, they will always have full measure, the best of its kind, and at a lower price than has ruled for a long time past. Ten per cent per ton will be saved on coal, if purchased within a few days, as the canals will soon be frozen, and the opportunity of obtaining it from boats will be lost.

